# Heterologously-expressed and Liposome-reconstituted Human Transient Receptor Potential Melastatin 4 Channel (TRPM4) is a Functional Tetramer

**DOI:** 10.1038/srep19352

**Published:** 2016-01-20

**Authors:** Maryrose Constantine, Chu Kong Liew, Victor Lo, Alex Macmillan, Charles G. Cranfield, Margaret Sunde, Renee Whan, Robert M. Graham, Boris Martinac

**Affiliations:** 1 Victor Chang Cardiac Research Institute, Lowy Packer Building, NSW 2010; 2 School of Medical Sciences, The Bosch Institute, The University of Sydney, NSW 2006; 3 Biomedical Imaging Facility, Lowy Cancer Research Centre, The University of New South Wales, Kensington, NSW 2052, Australia; 4 School of Life Sciences, University of Technology Sydney; 5 St Vincent’s Clinical School, University of New South Wales, Sydney, NSW 2010, Australia

## Abstract

Mutation, irregular expression and sustained activation of the Transient Receptor Potential Channel, type Melastatin 4 (TRPM4), have been linked to various cardiovascular diseases. However, much remains unknown about the structure of this important ion channel. Here, we have purified a heterologously expressed TRPM4-eGFP fusion protein and investigated the oligomeric state of TRPM4-eGFP in detergent micelles using crosslinking, native gel electrophoresis, multi-angle laser light scattering and electron microscopy. Our data indicate that TRPM4 is tetrameric, like other TRP channels studied to date. Furthermore, the functionality of liposome reconstituted TRPM4-eGFP was examined using electrophysiology. Single-channel recordings from TRPM4-eGFP proteoliposomes showed inhibition of the channel using Flufenamic acid, a well-established inhibitor of TRPM4, suggesting that the channels are functional upon reconstitution. Our characterisation of the oligomeric structure of TRPM4 and the ability to reconstitute functional channels in liposomes should facilitate future studies into the structure, function and pharmacology of this therapeutically relevant channel.

Ischaemic heart disease is currently the most common cause of mortality worldwide^1^,^2^. There is a pressing need for more effective pharmaceuticals and/or interventions to limit damage to the myocardium caused by reperfusion injury in patients with ischemic heart disease^1^,^2^,^3^. Recent studies in rodent models have shown that inhibition of TRPM4 abolished arrhythmias caused by ventricular hypoxia and re-oxygenation-induced early after depolarizations (EADs), or protected hearts subjected to global ischemia-reperfusion injury, where contractile function recovered significantly and infarct volume decreased^1^,^4^. Previous studies have also shown that 18 mutations in the TRPM4 gene are each linked to cardiac conduction disorders including progressive familial heart block type 1, isolated cardiac conduction disease, bifascicular block, atrio-ventricular conduction block and right bundle branch block^5^,^6^,^7^,^8^,^9^, indicating that this channel is critically involved in cardiac conduction^7^,^8^,^9^.

Cardiac conduction is closely related to mechano-electric feedback, which modulates heart rate^10^, and mechanosensitive ion channels detect mechanical stimuli such as atrial stretch^11^. Stretch of cardiac tissue can result in change of the shape and duration of action potentials, which in turn can lead to arrhythmogenesis^11^. TRPM4 has been suggested to function as a mechanosensitive channel^12^,^13^,^14^,^15^,^16^, but activation of TRPM4 by membrane stretch has only been demonstrated in a single study to date^17^. Whether TRPM4 is inherently mechanosensitive without the involvement of intermediate proteins or other cellular structures is currently unknown. The fact that multiple studies implicate TRPM4 as a potential pharmacological target for the treatment of heart failure and other cardiovascular diseases^4^,^5^,^6^,^18^,^19^ highlight the importance of obtaining a detailed understanding of TRPM4 structure and function (including confirmation of its mechanosensitivity).

The transmembrane domains of TRPM4 share high sequence similarity with those of other TRP channels^20^, and TRPM4 is expected to have a similar structure to the voltage and ligand-gated potassium channel superfamily^21^,^22^,^23^. Each TRPM4 monomer (or alpha subunit) is comprised of six transmembrane-spanning helical domains (S1-S6)^24^. The sensor region of TRPM4 has been reported to be located in helices S1-S4, and serves to receive and transduce signal to the gate that opens or closes the pore^5^,^24^. The selectivity pore is located between S5 and S6 and spans the lipid membrane, functioning to allow the selective passage of certain ions and/or hydrophilic molecules^5^,^7^,^13^,^24^. Mutagenesis studies of TRPM4 indicate that residues in the S5-S6 selectivity filter domain are critical in determining the conductance and ion selectivity of the channel^5^,^25^. The selectivity pore of TRPM4 is formed by the association of transmembrane domains from each subunit of the channel protein^5^,^24^. Using electron microscopy and cryo-electron microscopy, a number of other TRP channels, including TRPV1, TRPV4, TRPA1, TRPC3 and TRPM2, have been shown to form tetramers^26^,^27^,^28^,^29^,^30^. TRPM4 has also been predicted to form tetramers, which serve as the functional unit, though this has yet to be confirmed experimentally^5^. Recent evidence by Clemençon *et al.* 2014 suggests that TRPM4 is tetrameric, based on negative stain electron micrographs displaying low resolution particles of different size^31^. The majority of the projections are of TRPM4 in its monomeric form, while larger particles were identified as potential tetramers based on their overall size^31^. However, further studies are required to confirm this finding.

In this study we demonstrate that the supramolecular structure of TRPM4 is tetrameric, using a variety of biochemical methods. We also show that the liposome-reconstituted, heterologously expressed channel is functional and behaves similarly to TRPM4 endogenously expressed in human cell lines. Lastly, given that mechanosensitivity plays an important role in both normal physiology and cardiovascular diseases, including arrhythmia, hypertrophy and ischemia-reperfusion injury^11^, we also investigated the mechanosensitive properties of TRPM4 and determined that this channel does not appear to be intrinsically stretch modulated.

## Results

### TRPM4-eGFP expression and localisation

Expression of full length TRPM4 fused to eGFP was confirmed by Western blotting and a 473 nm laser scan of an SDS-PAGE gel. The presence of the FLAG tag, 8-Histidine tag and the C-terminal TRPM4 sequence was confirmed by Western blotting, which revealed a band of approximately 167 kDa in each blot (Fig. S1B–E). The presence of the eGFP tag in the fusion protein was confirmed by performing a 473 nm laser scan of the gel. A prominent band was detected at 167 kDa, which is in accordance with the expected size of the full-length monomeric protein (Fig. S1D).

Localisation of the expression of the TRPM4-eGFP protein in baculovirus-infected sf9 cells was determined using FM4-64 FX membrane staining and confocal microscopy. FM4-64 FX fluoresces strongly when bound to the outer leaflet of the plasma membrane and yields discrete plasma membrane staining^32^. As shown in Fig. S1F, expression of TRPM4-eGFP co-localised with the FM4-64 FX dye and is observed within the plasma membrane and around the perinuclear region of infected sf9 cells. The expression of the TRPM4-eGFP fusion protein in the plasma membrane of sf9 is consistent with previous studies visualising the location of hTRPM4 in human cells^7^,^33^,^34^,^35^.

### hTRPM4-eGFP fusion protein detergent screen

To estimate the size and homogeneity of the hTRPM4-eGFP fusion protein after solubilisation in different detergents, detergent-solubilised hTRPM4-eGFP samples were subjected to fluorescence size-exclusion chromatography (FSEC). Solubilisation of hTRPM4-eGFP with non-ionic detergents such as 1% LMNG, 1% digitonin, 2% DDM and 1% DM yielded mainly oligomeric protein, with the presence some of aggregates and degraded products (Figs S2 and S3). Solubilisation of hTRPM4-eGFP with 1% fos choline-14 resulted in 80% of the protein maintaining the oligomeric state, which was significantly higher than was observed with the other detergents. However, the overall yield of protein solubilised was much lower than for other detergents (Fig. S2). Ultimately, solubilisation hTRPM4-eGFP with 1% LMNG, 2% DDM or 1% digitonin was found to provide the highest yield of oligomeric protein and thus, TRPM4 was purified using mild non-ionic detergents.

### TRPM4-eGFP purification

The TRPM4-eGFP fusion protein was isolated under non-denaturing conditions and solubilised in n-dodecyl-β-D-maltopyranoside (DDM) and lauryl maltose neopentyl glycol (LMNG), which being mild detergents, are more likely to maintain membrane proteins in a native conformation compared to harsh ionic detergents^36^. Isolation of TRPM4-eGFP protein using Histidine affinity and size-exclusion chromatography yielded a sufficient amount of protein with adequate purity for downstream biochemical and biophysical characterisation. The UV and fluorescence chromatograms from the second round of size-exclusion purification displayed a large peak at 11-12 mL corresponding to oligomeric TRPM4-eGFP and a small peak at 8 mL (void volume) which contained aggregated protein (Fig. S4A,C). The protein from the fractions of the large peak at 11-12 mL were run on SDS-PAGE and then scanned for GFP fluorescence and/or Coomassie stained. The major band observed corresponded to a size of ~167 kDa (Fig. S4B,D). The identity of this band was confirmed as hTRPM4 using peptide mass fingerprinting analysis (Mass Spectrometry Core Facility (MCSF), Sydney University).

### Purification of cleaved TRPM4

After the first round of size-exclusion chromatography, the TRPM4-eGFP protein was cleaved with TEV protease to remove the eGFP-8His tag. TRPM4 was then exchanged into amphipol A8-35 and subjected to a second round of size-exclusion chromatography. The cleaved protein was dialysed into amphipol A8–35 to further stabilise the native protein structure in preparation for imaging by electron microscopy. The UV size-exclusion chromatogram of cleaved TRPM4 (without GFP tag) maintained in amphipol A8-35 contained a single, narrow, symmetrical peak (Fig. S4. E), which suggests that the protein was unlikely to be misfolded or aggregated. A fraction corresponding to the peak was analysed using SDS-PAGE and coomassie-stained, which revealed a band of ~137 kDa, indicating that the eGFP-8His tag was successfully cleaved off and that the protein was sufficiently pure (Fig. S4. F).

### Blue native PAGE

**Figure 1:**
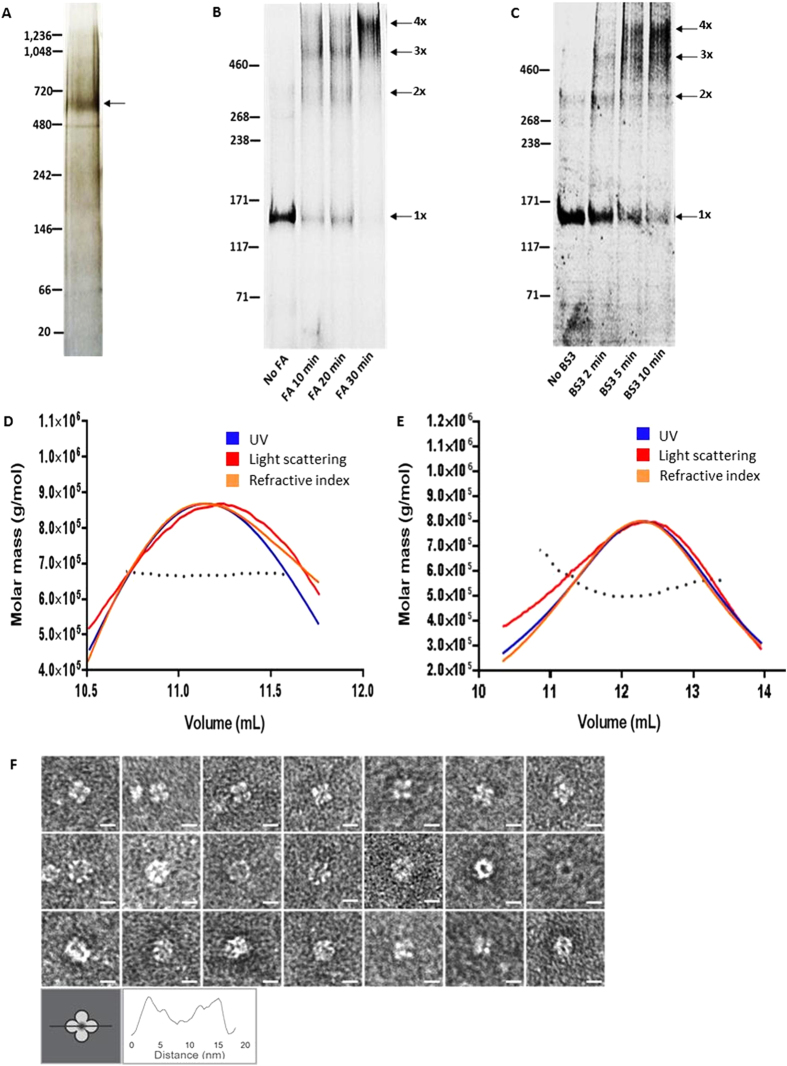
TRPM4 fusion protein forms tetramers. (**a**) Representative image of purified TRPM4 fusion protein (arrow) run on a silver stained native gel. (**b**) Crosslinking of purified TRPM4 fusion protein with formaldehyde (FA); samples were run on a denaturing tris acetate gel. The migration positions of the positions of monomeric (1×), dimeric (2×), trimeric (3×) and tetrameric (4×) species are indicated by arrows. (**c**) Crosslinking of hTRPM4-eGFP with *bis*-sulfosuccinimidyl suberate (BS3); samples were run on a denaturing tris acetate gel. The migration position of the monomeric and various crosslinked species are indicated as in (**b**). (**d**) SEC-MALLS chromatogram of the hTRPM4-eGFP fusion protein; molar mass (g/mol) of the protein was calculated from the light scattering, UV and refractive index signals. The black dotted line represents the fitting of the molar mass across the three signals. (**e**) SEC-MALLS chromatogram of cleaved TRPM4 protein, molar mass (g/mol) of the protein was calculated from the light scattering, UV and refractive index signals. The black dotted line represents the fitting of the molar mass across the three signals. (**f**) Transmission electron microscopy (TEM) images of cleaved hTRPM4-eGFP protein display a 4-fold symmetrical channel protein with a diameter of approximately 11–13 nm in the top panel (white scale bar is 10 nm). The middle and bottom panels of TEM images display side views of the channels or the electron-lucent pore ringed by protein. Schematic representation of the “4-leaf clover” shape and a representative cross-section profile are shown below.

Electrophoresis of TRPM4-eGFP using non-denaturing blue native PAGE resulted in a prominent band with a migration distance corresponding to an approximate M_r_ of 638,000/~638 kDa (Fig. 1A), which is ~3.8× the molecular weight of monomeric TRPM4-eGFP (167 kDa), suggesting that the protein is likely a tetramer. The addition of G-250 to the native gel is thought to coat the protein with a negative charge, thereby enhancing protein resolution, enabling more accurate size estimation. However, the conformation adopted by the native protein can cause ~15% error when estimating the expected size^37^.

### Crosslinking

To further confirm the oligomeric structure of TRPM4-eGFP, the channel was crosslinked using imidoester crosslinkers, FA or BS3. Imidoester crosslinkers initiate nucleophilic attack of lysine amino groups, which enables covalent binding of protein monomers via the crosslinker; however, the crosslinkers only bind surface residues that are already in close proximity in the native state^38^,^39^. The ability to isolate the same oligomeric species using multiple crosslinkers with different spacer arm lengths provides good evidence for protein subunits being in close proximity, reflective of an oligomeric state^39^. By increasing the duration of incubation of FA and BS3 with TRPM4-eGFP, three additional bands to the monomer band were formed. With the longest incubation times, TRPM4-eGFP appeared mostly as a single, high molecular weight band (Fig. 1B,C). Crosslinking of the TRPM4-eGFP fusion protein with formaldehyde (which has a short spacer arm of 2.3–2.77 Å) produced four bands, which are consistent with expected monomeric (167 kDa), dimeric (334 kDa), trimeric (501 kDa) and tetrameric (668 kDa) species of the protein (Fig. 1B). Likewise, crosslinking with BS3 which has a longer spacer arm (11.4 Å) also yielded bands indicative of the same sized species, which is further evidence that hTRPM4-eGFP forms a tetramer (Fig. 1C).

### SEC-MALLS of TRPM4-eGFP fusion protein and cleaved TRPM4 protein

To accurately determine the stoichiometry and molecular weight of the oligomers of TRPM4-eGFP as well as TRPM4 alone, the purified proteins were separated by size exclusion chromatography and the sizes of the eluted species were estimated using static light scattering (SEC-LS/UV/RI). The ASTRA analysis of the SEC-LS/UV/RI data for the hTRPM4-eGFP and cleaved TRPM4 is shown in Fig. 1D,E respectively. The molecular weights of TRPM-eGFP protein and cleaved TRPM4, calculated using the protein-conjugate analysis tool in the ASTRA 6 software, were determined to be 644.7 kDa (±0.336%) and 539.4 kDa (±1.899%). Given that the sizes of the monomers of the TRPM4-eGFP fusion protein and TRPM4 alone are 167 and 137 kDa respectively, the stoichiometry for both species is 4 monomers. The values calculated by the ASTRA 6 software are close to the expected values of 668 kDa for hTRPM4-eGFP and 548 kDa for free TRPM4, indicating that both proteins form a tetramer. The minor difference in the molecular weights calculated by the ASTRA software and the expected overall molecular weight for tetrameric hTRPM4-eGFP and cleaved hTRPM4 is not unexpected as there is usually over 5% error in determining the molecular weight of membrane proteins using SEC-MALLS^40^.

### Transmission electron microscopy of the cleaved hTRPM4 protein

Transmission electron microscopy was used to further characterise the stoichiometry, overall size, shape and architecture of cleaved (eGFP tag free) hTRPM4 protein. The electron microscopy images collected from hTRPM4 show distinct oligomers with a diameter of approximately 12 nm, which were observed in a range of orientations (Fig. 1F). The most distinct orientation displays a symmetrical “4 leaf clover” shape, suggesting tetrameric packing of the protein subunits (Fig. 1F, top panel). The diameter of the oligomeric TRPM4 measured from the particle images ranges from 11–13 nm. These values are similar to the diameter of the structure of TRPV1, determined using cryo-EM to be approximately 11 nm (in diameter) with fourfold symmetry^26^. The distinct tetrameric structure observed in some of the TRPM4 images is very similar to the top-down views of the TRPV1 channel obtained by Liao and co-workers (Fig. S5)^26^. A side-by-side comparison of the raw TEM images of TRPM4 and various class-average images of TRPV1 from previous studies^26^,^41^ highlights the striking resemblance between TRPV1 and TRPM4 in different orientations (Fig. S5) Thus, our electron microscopy data further confirms the data we have obtained from native gel electrophoresis, crosslinking and SEC-MALLS, which demonstrate that TRPM4 is tetrameric.

### Fluorescence lifetime Imaging (FLIM)

**Figure 2:**
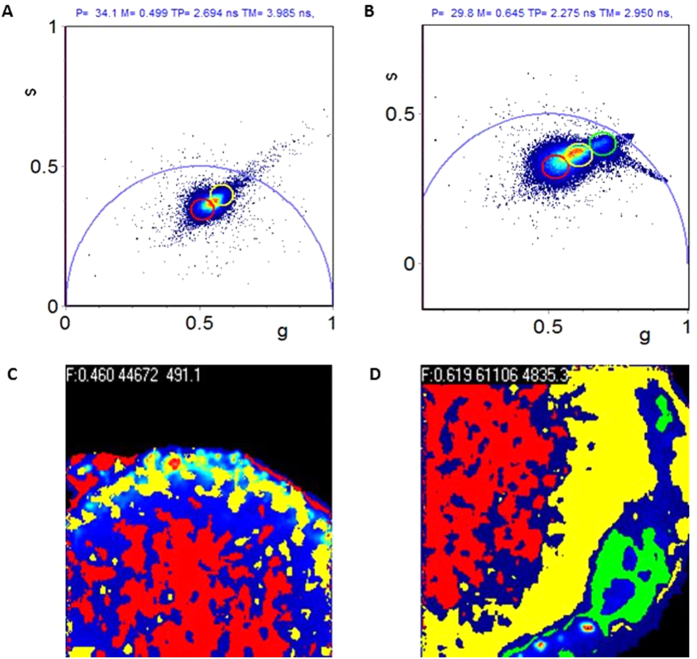
Fluorescence lifetime imaging (FLIM) of TRPM4 fusion protein incorporation into azolectin liposomes. (**a**) Phasor plot showing auto fluorescence phasor. (**b**) Phasor plot showing combination of autofluorescence and eGFP incorporated Phasor. (**c**) Phasor colour map of azolectin liposomes with no protein incorporated. Pixels in the image are colour coded according to the same coloured region of interest (ROI) selection in the phasor plot. (**d**) Fluorescence lifetime Phasor colour map of azolectin liposomes with TRPM4-eGFP fusion protein incorporated. The eGFP protein localisation is represented by green pixels in the image. All FLIM data were collected using time correlated single photon counting, and all phasors are shown in the 1^st^ harmonic (n = 6).

Having determined that purified hTRPM4 likely forms a tetramer, which suggested that it may be functional, we reconstituted the channel into liposomes in order to investigate its functional properties. We selected soybean azolectin as the lipid for reconstitution of hTRPM4-eGFP as it is a mixture of lipids, which has been frequently used for studies of mechanosensitive channels and that allows the protein to select appropriate surrounding annular lipid molecules, which enabled reconstitution of the protein into liposomes^42^. To confirm that hTRPM4-eGFP was successfully reconstituted into the liposomes, we used fluorescence lifetime imaging (FLIM). When soybean azolectin is excited by a 488 laser on a confocal microscope it appears to be fluorescent^43^; thus to differentiate between azolectin liposomes and TRPM4-eGFP proteoliposomes we used fluorescence lifetime imaging as the lifetime of eGFP is shorter than that of azolectin alone. The FLIM data were analyzed in the Phasor plot by selecting clusters of pixels, with different lifetime clusters differentiated by colour selection (Fig. 2A–D). The phasor approach is advantageous because it is a simple way to analyse FLIM data and the problems associated with exponential analysis are avoided by providing a graphical global view of the processes which influence fluorescence decay at individual pixels^44^. Since different molecular species have a specific phasor, individual molecules may be identified via their location on the phasor plot^44^. The hTRPM4-eGFP protein incorporated into azolectin liposomes/lipid has a phasor position shifted from that of the autofluorescence component of azolectin alone, indicating a difference in the fluorescence lifetimes of the two species (Fig. 2A,B). Encapsulated eGFP is highlighted by the green cursor on the phasor and its position is highlighted in the intensity image. It appears that the hTRPM4-eGFP protein is not evenly distributed throughout the azolectin lipid where the protein has inserted itself into the membrane in clusters (Fig. 2D). Thus, hTRPM4-eGFP was successfully reconstituted into azolectin lipid and liposomes, as indicated by the difference in the lifetimes of the two species. This finding provided the justification for further functional characterisation of the fusion protein in proteoliposomes, using patch clamp electrophysiology.

### Functional properties of hTRPM4-eGFP incorporated into proteoliposomes

**Figure 3:**
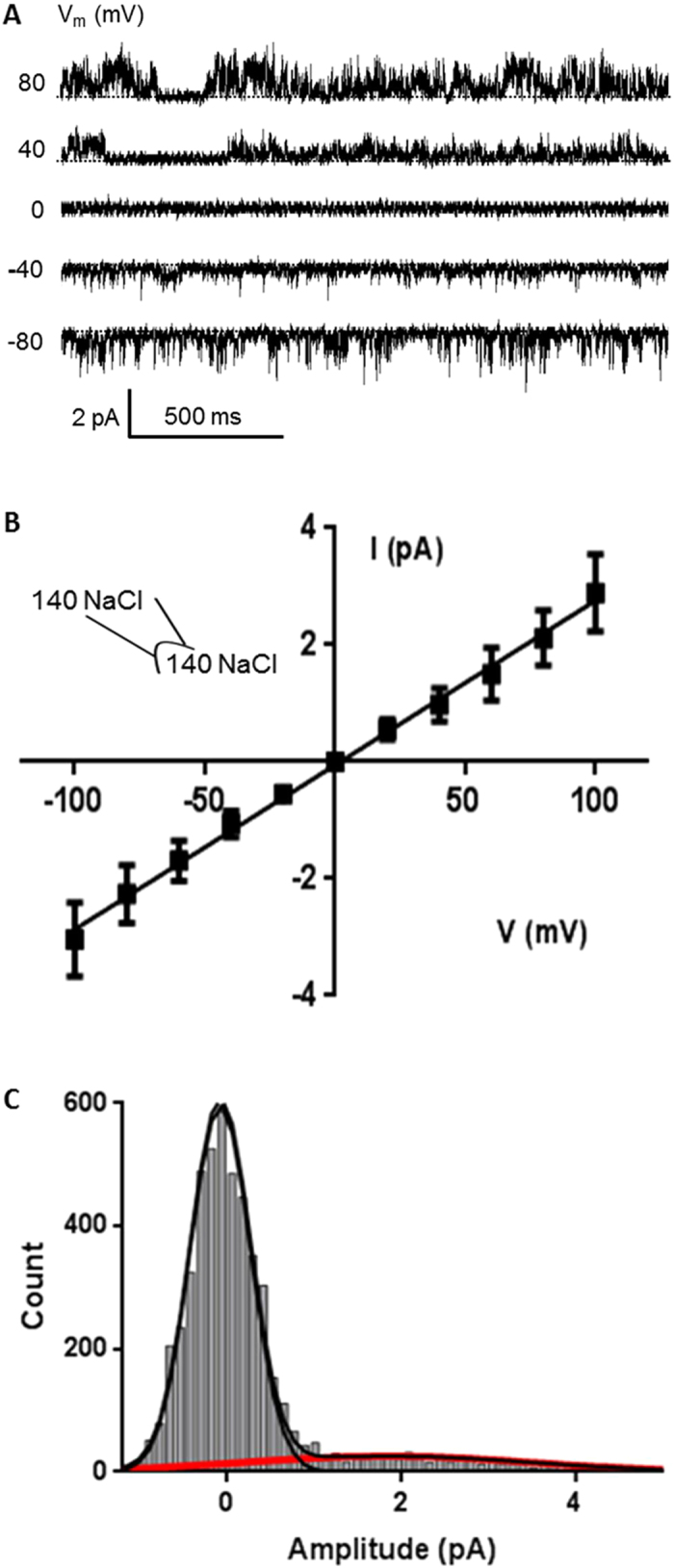
Conductive properties of hTRPM4-eGFP. (**a**) Single channel traces recorded at different voltages from an excised inside-out patch. Pipette contained 140 mM NaCl, 5 mM KCl, 10 mM CaCl_2_ and 5 mM HEPES pH 7.5. V_m_ represents the membrane potential. (**b**) Current (I)-voltage (V) relationship determined under the same ionic conditions as in (**a**) (n = 7). (**c**) Amplitude histogram of hTRPM4-eGFP at 100 mV, using the same ionic conditions as in (**a**).

To determine if purified hTRPM4-eGFP was functional and possessed similar channel properties to TRPM4 overexpressed in mammalian cells (HEK293 cells) the activity of the hTRPM4-eGFP incorporated into liposomes was characterised using patch clamp electrophysiology. Under symmetric bath conditions (0.14 M NaCl, 0.005 M KCl) the current-voltage (I-V) relationship for the hTRPM4-eGFP channel showed that the single channel current was ohmic between –100 mV and +100 mV, with a slope conductance of approximately 28 ± 1 pS (Fig. 3A,B, n = 7). These results indicate that hTRPM4-eGFP had a similar conductance to that of TRPM4 expressed in human cell lines^45^,^46^,^47^. Amplitude histogram analysis and kinetic modelling of the single channel recordings from hTRPM4-eGFP in proteoliposomes indicated that the fusion protein has a similar conductance of 24 pS (Fig. 3C) and the same number of open and closed states as TRPM4 expressed in HEK293 cells, which has two closed states and one open state (Fig. S6B-C)^48^.

**Figure 4:**
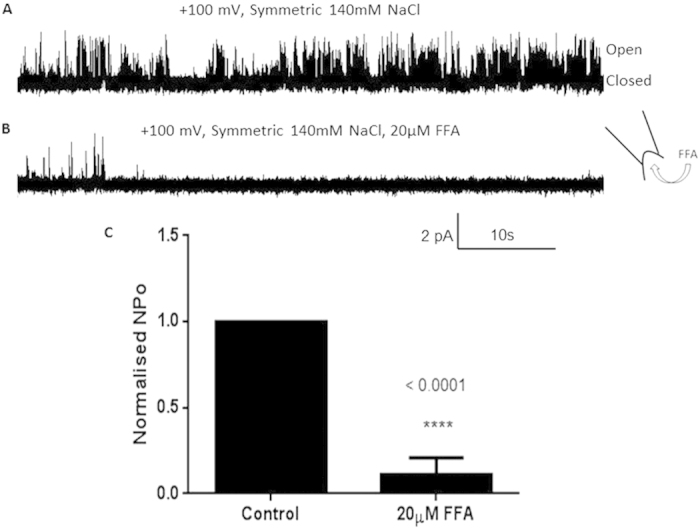
hTRPM4-eGFP inhibition by Flufenamic acid. (**a**) Single channel trace of hTRPM4-eGFP recorded from an excised inside-out patch. Pipette and bath contained 140 mM NaCl, 5 mM KCl, 10 mM CaCl_2_ and 5 mM HEPES pH 7.5. (**b**) Single channel trace of hTRPM4-eGFP in the presence of 20 μM flufenamic acid (FFA). Pipette and bath contained the same solution as in (**a**). (**c**) Normalised open probability histogram of hTRPM4-eGFP before and after FFA (20 μM) addition (n = 5, p < 0.0001).

Recordings were also obtained in the presence of 20 μM flufenamic acid (FFA) to ensure functional selection^4^. The channel activity was inhibited when 20 μM FFA was added to the bath (Fig. 4A,B) causing the open probability Po to decrease significantly (Fig. 4C). The fact that the hTRPM4 fusion protein displayed sensitivity to FFA indicates that heterologously expressed TRPM4 reconstituted into liposomes is functional and behaves similarly to TRPM4 expressed in human cell lines. Since the hTRPM4 fusion protein responded to FFA added to the bath, it suggests that the intracellular portion of the channel protein uniformly faces the bath, in line with what has been reported for liposome reconstituted mechanosensitive channels MscL and MscS^49^,^50^. The efficiency of obtaining patches containing functional channels was very low (approximately 1% success rate). This is not surprising given that TRPM4 is a large, transmembrane protein with 6 extracellular loops, making it likely that most of the protein molecules were only partially reconstituted and/or inserted in liposomes which precluded proper channel function. A study by Dorr *et al.* in 2014, used detergent-free isolation of KscA from E.coli using native nanodiscs to reconstitute the protein into planar lipid bilayers^51^. Such an approach may improve the likelihood of reconstituting functional TRPM4 in the future given the more favourable lipid environment. Another possible reason for the low efficiency in obtaining functional channels is that the incorporation of the protein into liposomes was not evenly distributed as shown in Fig. 2D, where the protein inserted in clusters of certain regions of liposomes. Therefore, it was challenging using conventional path-clamp electrophysiology equipment to ascertain which regions of the liposome the protein had been incorporated into.

**Figure 5:**
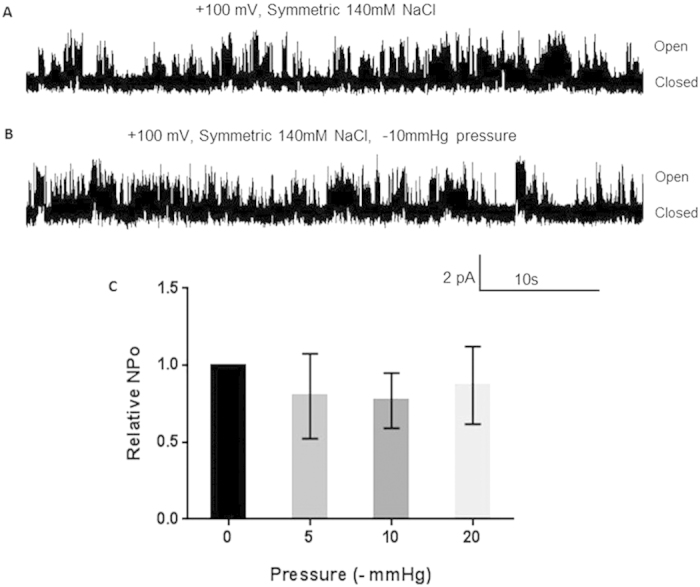
hTRPM4-eGFP is not affected by membrane stretch. (**a**) Representative single channel trace of hTRPM4-eGFP without stretch applied to the patch. Pipette and bath contained 140 mM NaCl, 5 mM KCl, 10 μM CaCl_2_ and 5 mM HEPES pH 7.5. (**b**) Representative single channel trace of hTRPM4-eGFP with –10 mmHg stretch applied to the patch. Pipette and bath contained the same solution as in (**a**). (**c**) Relative open probability histogram of hTRPM4-eGFP under varying amounts of stretch (0, −5, −10 and −20 mmHg) (n = 3–4, p > 0.05).

Morita *et al.*, 2007 demonstrated that hTRPM4 overexpressed in HEK293 cells exhibited mechanosensitivity in cell-attached patches upon application of -20 mmHg stretch^17^. In this study the purified hTRPM4-eGFP fusion protein was reconstituted into azolectin liposomes to determine whether TRPM4 is intrinsically stretch-activated or mechanosensitive. Purified channels reconstituted into liposomes are a useful models for confirming whether a channel protein is intrinsically stretch-modulated due to the absence of other background channels and cellular components, which may participate in the transmission of mechanical stimuli. In our study, TRPM4 did not appear to be inherently mechanosensitive given that the activity of the channel protein remained unaffected by membrane tension despite subjecting the liposome patches to various degrees of membrane stretch (i.e. −5, −10 and −20 mmHg; Fig. 5A,B) and observing no difference in open probability Po (Fig. 5C). A similar result was also observed for the dmPiezo1 channel; Coste and colleagues reconstituted dmPiezo1 into planar bilayers, resulting in constitutively active channels^37^ suggesting that planar bilayers and patches were potentially stretched close to the bilayer lytic tension^52^, making the channels apparently non-responsive to changes in mechanical stress. It is possible that the lack of a cytoskeleton, asymmetry of the monolayer lipid composition of the membrane bilayer and channel clustering are important for TRPM4 mechanosensitivity, with the absence of all of these factors in liposomes accounting for the observed lack of responsiveness to stretch when reconstituted in liposomes^53^. Additionally, it has been shown that TRPM4 couples to mechanosensitive purinergic receptors P2Y4 and P2Y6, which modulate TRPM4 activity to mediate pressure-induced depolarisation and myogenic constriction in cerebral parenchymal arterioles^54^. Future studies should clarify the origin of TRPM4 mechanosensitivity by varying the lipid composition in liposomes^43^, adding amphipatic molecules such as LPC to liposomes^55^, patching TRPM4 in cellular blebs^56^ or including known interacting TRPM4 binding partners such as purinergic receptors, which can detect and transmit membrane tension.

## Discussion

In this study, we have provided substantial evidence to support the view that TRPM4 forms tetrameric ion channels, which can be functionally reconstituted into liposomes. Furthermore, our work suggests that TRPM4 channels are not inherently mechanosensitive, i.e. they cannot by activated by stretching the lipid bilayer alone, but may require additional membrane components including cytoskeleton, extracellular matrix and/or interaction with other membrane proteins to exhibit mechanosensitivity. In summary, hTRPM4-eGFP was overexpressed in sf9 cells, the channel protein was purified and the oligomeric form of the channel protein was determined using SEC-MALLS, crosslinking, native gel electrophoresis and TEM. This work is the first to convincingly demonstrate that the oligomeric structure of TRPM4 is tetrameric, using a variety of methods. Additionally, the negative stain TEM images we have acquired of TRPM4 are the first to display its structure in detail. The electron micrographs we have obtained bear significant resemblance to the class average images of TRPV1 derived from TEM and cryo-electron microscopy. The elucidation of the oligomeric structure of TRPM4 and the procedure for obtaining workable amounts of recombinant TRPM4 reported in this study should facilitate future investigation into the structure and function of TRPM4.

Additionally, the procedures described in this study have enabled the functional properties of the hTRPM4-eGFP channel to be studied in liposome bilayers. The incorporation of the purified hTRPM4-eGFP protein into azolectin liposomes was confirmed using Fluorescence lifetime imaging. Fluorescence lifetime imaging may also be used in future studies to assess whether other fluorescently labelled ion channels are incorporated into lipid bilayers or liposomes for liposomal assays or functional studies. The ability to characterise the gating properties of purified TRPM4 channels in artificial bilayers should also prove useful in future studies of the structure–function relationships and the pharmacology of this ion channel. By varying the bilayer composition, it should be possible to obtain detailed information on protein-lipid interactions between TRPM4 and certain types of lipids, i.e. their head groups as well as acyl chains. Lipid bilayer variation will also facilitate the determination of the effect of lipid ordering on channel gating. To our knowledge this is also the first work to demonstrate successful functional reconstitution of purified TRPM4 protein into exogenous lipid bilayers, which may greatly assist the evaluation of future pharmacological agents targeting this therapeutically relevant ion channel.

## Materials and Methods

### FLAG-hTRPM4-eGFP-8His expression

The human *trpM4b* sequence was amplified using the sequence and ligase independent cloning (SLIC) primers shown below:

h*trpM4b* Fwd Prim: 5′-CAGGGGCCCCAAAGGCCTGTGGTGCCGGAGAAGGAG-3′

htrpM4b Rev Prim: 5′-GCTCGTCGACGTAGGCCTGTCTTTGGACCCAGGCAGGTCA-3′

The pFBDual FPLR_TEVGFPHIS vector (GeneArt) encoding the proteins indicated (shown in #1, below) was digested with Stu I and then purified (Promega PCR clean up kit):

### FLAG- < protein of interest > -TEVsite-GFP-8His = 7.3 kb

*htrpM4* was cloned into the vector using a ligation independent cloning (EcoDry In-fusion reactions, Clontech), method, as described in In-Fusion HD EcoDry Cloning kit user manual. Recombinant baculovirus was then generated using the manufacturer’s protocol (Bac-to-Bac expression system, Invitrogen). To express the protein, *Spodoptera frugiperda* (sf9) cells were infected with recombinant baculovirus and harvested 72-80 h thereafter. Cells were flash frozen and stored at −80 °C or used immediately. To determine the localisation of the TRPM4-eGFP fusion protein expression, baculovirus infected sf9 were co-stained with FM4-46FX lipophylic styryl dye and imaged using a confocal microscope, see *SI text* for details.

### Western blotting

Expression of the full length fusion protein was confirmed by Western blotting. Membranes were probed with FLAG M2, TRPM4 and His antibodies, see *SI Materials and Methods* for more detail.

### Gel fluorescence scan

The presence of the GFP tag on the C-terminus of the protein was confirmed by performing a 473 nm laser scan of the gel. Crude lysates of TRPM4-eGFP baculovirus infected sf9 and/or purified TRPM4-eGFP fusion protein were mixed with 5× Laemmli loading buffer and then subjected to SDS-PAGE gel (TGX 4–12% Bio-Rad) fractionation. Subsequently the gel was scanned using the 473 nm laser line on Fujifilm Image Reader FLA-5000 V1.0. The data was processed using Multi Gauge 2.3 software.

### Detergent Screen for the TRPM4-eGFP fusion protein

A detergent screen was performed for the hTRPM4-eGFP fusion protein, where 5 mL pellets of frozen baculovirus infected cells were solubilised in several different detergents including 1% Fos-choline-14, 2% DDM, 1% DM, 1% Lauryl Maltose Neopentyl Glycol (LMNG), 1% digitonin and a mixture of 0.5% DDM plus 0.5% LMNG (wt/vol) dissolved in a base buffer containing (40 mM Tris pH 7.5, 150 mM NaCl, 150 mM KCl, 1 mM MgCl2, 2 mM CaCl2, 6 mM β-mercaptoethanol, 5 mM L-Arginine and 10% glycerol). All cell pellets were solubilised for 2 h at 4 °C with rotation before being subjected to fluorescence size-exclusion chromatography (FSEC) to determine the size homogeneity of the proteins after solubilisation (whether they be aggregated, oligomeric or degraded). FSEC was performed on an AKTA purifier system (Amersham Biosciences) using a Superose 6 10/300 GL column (GE Healthcare) pre-equilibrated with the base buffer and 0.01% (wt/vol) of the detergent the cell pellet was solubilised in. 1 mL of the solubilised material was injected per run onto the Superose 6 column and fractions were collected. Samples from individual fractions (80 μL each) were aliquoted into in a black 96 well microplate (Corning) and scanned for fluorescence using a BMG PHERAstar FS multimode plate reader (BMG Labtechnologies, Durham, NC).

### TRPM4-eGFP Protein purification

Cells were resuspended in a Buffer containing 1.5% DDM (Anatrace) or 0.5% LMNG (Anatrace) and the suspension was dounce homogenised then solubilised for 2 h with rotation at 4 °C. After solubilisation, cell debris was cleared by low-speed centrifugation (5000 g × 20 min) and the supernatant fraction was purified using His-tag affinity and fluorescence size exclusion chromatography (FSEC), see *SI Materials and Methods* for detail. The identity of the TRPM4 sequence was confirmed using MALDI-TOF peptide mass fingerprinting (Sydney Proteome Group, Sydney University).

### SEC-MALLS

The molecular mass of oligomeric TRPM4-eGFP and cleaved TRPM4 were determined using a miniDAWN TREOS three-angle static-light scattering detector and a WYATT refractive index detector (Wyatt Technology, Santa Barbara, CA). Size-exclusion purified TRPM4-eGFP (500 μL) was injected onto the Superose 6 column (GE Healthcare Life Sciences, pore size 400 Å and protein MW range 5–5000 kDa) pre-equilibrated in running buffer A (pH 7.8) containing 0.1% DDM, and eluted at a rate of 0.5 mL/min on the Wyatt SEC-MALLS system. Alternatively, size exclusion purified cleaved TRPM4 (500 μL) in amphipols was injected onto a Superose 6 column equilibrated in running buffer A without detergent at 0.5 mL/min using the Wyatt SEC-MALLS system. Refractive index and light scattering detectors were calibrated following the manufacturer’s guidelines. The data were processed and analysed using the protein conjugate template of the ASTRA 6.1.2 software (Wyatt Technology). See *SI text* for more detail.

### Native gel electrophoresis

To determine the overall size of hTRPM4-eGFP, the purified fusion protein was fractionated on a non-denaturing NativePAGE Novex 3–12% Bis-Tris protein gel (1 mm, 10 well) using the Novex NativePAGE Bis-Tris system (Life technologies), as per manufacturer’s protocol. Briefly, the TRPM4-eGFP was mixed with NativePAGE sample buffer and NativePAGE 5% G-250 Sample Additive and then fractionated at 150 V for 2 h. Lastly, the native gel was silver stained using SilverQuest Silver Staining Kit (Invitrogen) according to the manufacturer’s protocol.

### Formaldehyde and BS3 Crosslinking

TRPM4-eGFP was crosslinked using the imidoester crosslinkers formaldehyde (FA, Sigma-Aldrich) and *bis*-sulfosuccinimidyl suberate (BS3, Thermo Fisher Scientific). Purified TRPM4-eGFP protein was incubated with or without 0.2% formaldehyde (FA) at room temperature for varying time points (10 min, 20 min and 30 min). Additionally purified TRPM4-eGFP was incubated with or without 2.6 mM *bis*-sulfosuccinimidyl suberate (BS3) for different times (2 min, 5 min and 10 min). After incubation of the samples with their respective crosslinker for various times, NuPAGE LDS sample buffer and NuPAGE Reducing Agent (Invitrogen) were added and mixed. The samples were then denatured at 70 °C for 10 min before performing electrophoresis using a 3–8% NuPAGE Tris-Acetate gel under denaturing conditions according to manufacturer’s instructions. Gels were imaged using a Fujifilm Image Reader FLA-5000 V1.0 (473 nm laser line) and data processed using Multi Gauge V2.3 software.

### Electron Microscopy

Carbon-coated 200 mesh copper TEM grids (ProSCiTech, Australia) were rendered hydrophilic by exposure to UV light (254 nm) for 30 min and then floated on the surface of a drop of cleaved, purified TRPM4 (10 uL at 50 μg/μL) for 1 min. Excess sample was removed from the grids by contact with filter paper. The grids were then washed by floating briefly on a 10 μL drop of filtered MilliQ water and blotted dry. The samples were negatively stained by floating on a 20 μL drop of 2% wt/vol uranyl acetate for 2 min, followed by filter paper blotting. EM grids were imaged on a Phillips CM120 Biofilter TEM, operating at 120 kV at the Australian Centre for Microscopy and Microanalysis, University of Sydney. Images were recorded at a magnification of 55,000× using a 1024 × 1024 post column Gatan Imaging Filter (GIF) camera.

### Fluorescence Lifetime Imaging

Fluorescence lifetime imaging (FLIM) was used to demonstrate that hTRPM4-eGFP was incorporated into azolectin liposomes/lipid, see *SI text* for the method of protein incorporation into liposomes. When soybean azolectin is excited by a 488 laser on a confocal microscope it appears to be fluorescent^43^; thus to differentiate between azolectin liposomes and TRPM4-eGFP proteliposomes we used fluorescence lifetime imaging since the lifetime of eGFP is shorter than that of azolectin alone. A 10 μL aliquot of rehydrated liposomes was placed in a Fluorodish (WPI, FD35-100) and diluted with 200 μL buffer containing 140 mM NaCl, 5 mM KCl, 0.1 mM CaCl_2_ and 5 mM HEPES, pH 7.5. FLIM images were acquired on a PicoQuant MicroTime 200 inverted confocal microscope using a 60×, 1.2-NA water immersion objective at the University of NSW Biomedical Imaging Facility (BMIF). Samples were pulsed at 470 nm with a fiber-coupled, pulsed laser diode with a pulse width under 200 ps. A single-photon avalanche diode (SPAD, microphoton devices) detected signals in the range of 489–532 nm, which was connected to time-correlated single-photon counting (TCSPC) electronics (PIcoHarp 300; PicoQuant). FLIM data were analysed by a phasor approach^44^ as described in *SI text*, using SimFCS software developed at the Laboratory for Fluorescence Dynamics (www.lfd.uci.edu).

### Electrophysiology

An aliquot (3 uL) of rehydrated liposomes was added to a patch clamp chamber filled with recording solution containing 140 mM NaCl, 5 mM KCl, 10 μM CaCl_2_ and 5 mM HEPES pH 7.5. Pipettes were made from borosilicate glass (Sigma-Aldrich) and pulled using a micropipette puller (Narashige, Japan) to an average resistance of 8-10 MΩ. Both the pipette and bath were loaded with the same recording solution. Gigaohm seals were obtained after applying suction with the pipette to a unilamellar blister. Episodic voltage ramps were applied from −100 mV to +100 mV over 2.5 sec. Single channel events were recorded at +/−80 mV and +/−100 mV from excised inside-out patches. After obtaining single channel activity, 20 μM of FFA, an inhibitor of TRPM4 channels, was added to the bath. Stretch was also applied to the membrane patch using a syringe to apply suction to the patch pipette. The pressure applied was detected by a pressure monitor (World Precision Instruments, WPI) or High Speed Pressure Clamp (ALA Scientific instruments). An AxoPatch 200B amplifier (Axon Instruments) was used to amplify currents and data acquired at 5 kHz sampling rate with 2-kHz filtration. Data were acquired using pClamp 10.2 software, and then analysed using Clampfit 10.2 (Axon Instruments, CA) and QuB version 2.0.0.22 software (University of Buffalo). Statistical significance was determined using an unpaired t-test (GraphPad Prism 6 software).

## Additional Information

**How to cite this article**: Constantine, M. *et al.* Heterologously-expressed and Liposome-reconstituted Human Transient Receptor Potential Melastatin 4 Channel (TRPM4) is a Functional Tetramer. *Sci. Rep.* **6**, 19352; doi: 10.1038/srep19352 (2016).

## Supplementary Material

Supplementary Information
