# Supplementary material for: Heterologously-expressed and Liposome-reconstituted Human Transient Receptor Potential Melastatin 4 Channel (TRPM4) is a Functional Tetramer
Source: Sci Rep. 2016 Jan 20;6:19352. doi: 10.1038/srep19352 (PMC4726259; doi:10.1038/srep19352)
Supplement: Supplementary Information [file srep19352-s1.docx]

**Supporting Information (SI)**

**SI Materials and Methods**

**Confocal Imaging of TRPM4-EGFP baculovirus infected sf9 co-stained with FM4-46FX Lipophylic Styryl dye**

Confocal imaging was used to determine the localisation of the TRPM4-eGFP fusion protein expression in baculovirus infected sf9. Firstly, a stock solution of the lipophilic probe membrane stain FM4-64FX (Life Technologies, F34653) was prepared by dissolving 1µg/1µL of the dye in DMSO. Then the stock solution was diluted to 5µg/mL of FM4-64FX in 1 x PBS which was kept chilled on ice. Subsequently, a 1mL suspension culture of M4-EGFP infected cells was harvested (72-80 hrs post infection), and then pelleted by centrifugation at 800g for 2 min at 4°C. The supernatant was removed and 100µL of FM4-64FX working solution was added to the cell pellet, resuspended and kept on ice. The suspension was then placed in a Fluorodish (WPI, FD35-100) just prior to imaging on the confocal microscope. Imaging was performed simultaneously using a Zeiss LSM700 inverted confocal microscope, 63 x water objective 1.0 NA (488- and 594-nm diode laser lines).

**Western Blotting**

Purified protein (~50 μg) was mixed with 5 x Laemmli dye (4:1) and then fractionated on a pre-cast SDS-PAGE gel (TGX 4-12%, Bio-Rad, Hercules, CA) for 40 mins at 200V (Bio-Rad Mini-PROTEAN). Fractionated proteins were then transferred onto a PVDF membrane using an Invitrogen wet transfer cassette at 35mV for 1h. The membrane was washed briefly with Tris-Buffered Saline Tween-20 buffer (TBST) and then blocked with 5% skim milk in TBST for 1h at room temperature with rotation. The membrane was then incubated with the following primary antibodies separately: anti-FLAG M2 (N-term) mouse monoclonal antibody (1:3000, Sigma Aldrich, F1804), anti-TRPM4 (C-term) rabbit polyclonal antibody (1:2000, ab63080) and anti-His (C-term) mouse monoclonal antibody with Alkaline Phosphatase Conjugate (1:6000, Life technologies, R932-25), in a diluent of 1% skim milk in TBST. The membrane was incubated with the primary antibodies for 1h at room temperature, then overnight at 4°C with mixing/rotation. Following this, the membrane was washed with TBST and then probed with secondary antibodies (1:8000 goat anti-rabbit IgG-HRP, Thermo Fisher Scientific; or 1:5000 ECl Anti-Mouse IgG-HRP from sheep (GE healthcare NA931V) in 1 % skim milk in TBST diluents. The secondary antibodies were incubated with the membrane for 2h at room temperature with rotation. Finally the membrane was washed with TBST and proteins detected using SuperSignal West Pico Chemiluminescent substrate ECL (Thermo Fisher Scientific, 34080).

**FLAG-hTRPM4-eGFP-8His Protein purification**

Cells were resuspended in Buffer A (150mM NaCl, 150mM KCl, 25mM disodium hydrogen orthophosphate, 1.8mM potassium dihydrogen orthophosphate, 5mM L-arginine, 10% glycerol pH7.5 adjusted with HCl) containing 1.5% DDM (Anatrace) or 0.5% LMNG (Anatrace) and supplemented with an EDTA (ethylenediaminetetraacetic acid) -free protease inhibitor cocktail tablet (Roche). The detergent to protein ratios for DDM and LMNG were 1:4.6 and 1:13.8 respectively. The suspension was homogenised using 20 strokes of a Dounce homogenizer, the protein was solubilised for 2h with rotation at 4°C. After solubilisation, cell debris was cleared by low-speed centrifugation (5000g x 20min) and then the supernatant fraction was incubated with cobalt talon resin (Clontech) for 2h with rotation at 4°C. Following this the resin was pelleted by low speed centrifugation (300xg, 5min) and washed with Buffer A containing 0.1% DDM or 0.01% LMNG and 5mM Imidazole. The protein was then eluted from the resin with Buffer A containing 0.1% DDM or 0.01% LMNG and 250mM imidazole. One mL of buffer was applied at a time to collect concentrated elutions in 1mL aliquots. The fluorescence of the histidine affinity-tag-purified protein fractions was determined using a BMG PHERAstar FS multimode plate reader (BMG Labtechnologies, Durham, NC) and 96 well black microplates (Corning). Subsequently, the most highly fluorescent fraction eluted from the His-affinity matrix was subjected to size-exclusion chromatography (SEC) on an AKTA purifier system (Amersham Biosciences) using a Superose 6 10/300 GL column (GE Healthcare) equilibrated with Buffer A.

Alternatively, after His-affinity purification TRPM4-eGFP protein was dialysed against buffer A and then the C-terminal EGFP-8His tag was proteolytically cleaved by incubation with TEV protease for 30 min at room temperature, followed by overnight incubation with rotation at 4˚C. Cleaved TRPM4 was incubated with the amphipathic surfactant, Amphipol A8-35 (Anatrace) at 1:3 (wt/wt) with mixing overnight at 4˚C. Bio-Beads SM2 adsorbants (15mg, Bio Rad) were added to the sample and the sample mixed gently for 4h at 4˚C to scavenge excess detergent. Subsequently, the sample was subjected to size-exclusion/gel filtration chromatography (as described above) in Buffer A without detergent added.

The size homogeneity of samples was assessed by fluorescence size exclusion chromato-graphy (FSEC), where aliquots of each fraction from SEC were placed in a black 96 well microplate (Corning) and scanned for fluorescence using a BMG PHERAstar FS multimode plate reader (BMG Labtechnologies, Durham, NC). The identity of the TRPM4 sequence was confirmed using MALDI-TOF peptide mass fingerprinting (Sydney Proteome Group, Sydney University).

**SEC-MALLS**

The dn/dc values used for the analysis were 0.185mL/g for proteins, 0.1435mL/g for DDM detergent (Anatrace) and 0.1424mL/g for amphipol A8-35. The dn/dc value for amphipol A8-35 was determined according to the method outlined in Strop et al. 2005. Different concentrations of A8-35 (0.044, 0.092, 0.138, 0.184, 0.28, 0.368 and 0.46mg/mL) were run twice in batch (determine dn/dc) mode on an Optilab rEX refractometer (Wyatt Technology, Santa Barbara, CA) and data were analysed with Astra 6.1.2 software, for the linear regression r^2^ =0.999. The extinction coefficient used for DDM was 0.0044, the extinction coefficient for amphipol A8-35 was determined to be 0.0386 at 280nm. While the extinction co-efficients for TRPM4-eGFP and cleaved TRPM4 at 280nm were calculated using the ProtParam tool on the ExPasy server (expasy.org) to be 1.297 mL g^−1^ cm^−1^ and 1.409 mL g^−1^ cm^−1^, respectively.

**Preparation of proteoliposomes**

Functionality of the protein was assessed by patch-clamp recording after reconstitution into liposomes. Soybean azolectin lipid (Avanti) was dissolved in chloroform at 10mg/mL. The lipid (2mg) was dehydrated under a Nitrogen stream and then resuspended in 1mL Dehydration-Rehydration (D-R) buffer (200mM KCl, 5mM HEPESm pH7.2) by vortexing and sonication until the solution looked cloudy and uniform. Purified hTRPM4-eGFP was mixed with the lipid suspension at a ratio of 1:50 or 1:200 for 1h with rocking at 4°C. To remove the detergent, Biobeads SM2 (Bio-Rad) (15mg/mL) were added and the mixture incubated for 3h at 4°C. The supernatant was collected and ultracentrifuged at 43,000rpm for 30min (50.2Ti, Beckman) to collect the proteoliposomes. The supernatant was removed and the pellet resuspended in 50µL of D-R buffer and aliquots of the liposomes were placed on glass slides and dehydrated overnight at 4°C in a dessicator. The following day the dried liposomes were rehydrated with 20uL D-R buffer for 24hrs at 4°C in a petri dish containing a sheet of wet filter paper, before being used for imaging and/or patch-clamp recording.

**Fluorescence Lifetime Imaging Analysis**

In phasor analysis of FLIM data, the fluorescence lifetime decays are Fourier transformed and the data represented in a 2D plot of S (imaginary part) versus G (real part) of the Fourier transformation, as previously described [^1^](#_ENREF_1). The resulting position in the phasor plot represents the average lifetime. The *s* and *g* component are given by the following expressions:

$$s(\omega)=\frac{\int_{0}^{\infty} I\left( t \right)sin(\omega t)dt}{\int_{0}^{\infty} I\left( t \right)dt}$$

$$g(\omega)=\frac{\int_{0}^{\infty} I\left( t \right)\cos\left( \omega t \right)dt}{\int_{0}^{\infty} I\left( t \right)dt}$$

Where *f* is the laser repetition rate, *ω=2πf* and *I(t)* is the fluorescence intensity at time *t.*


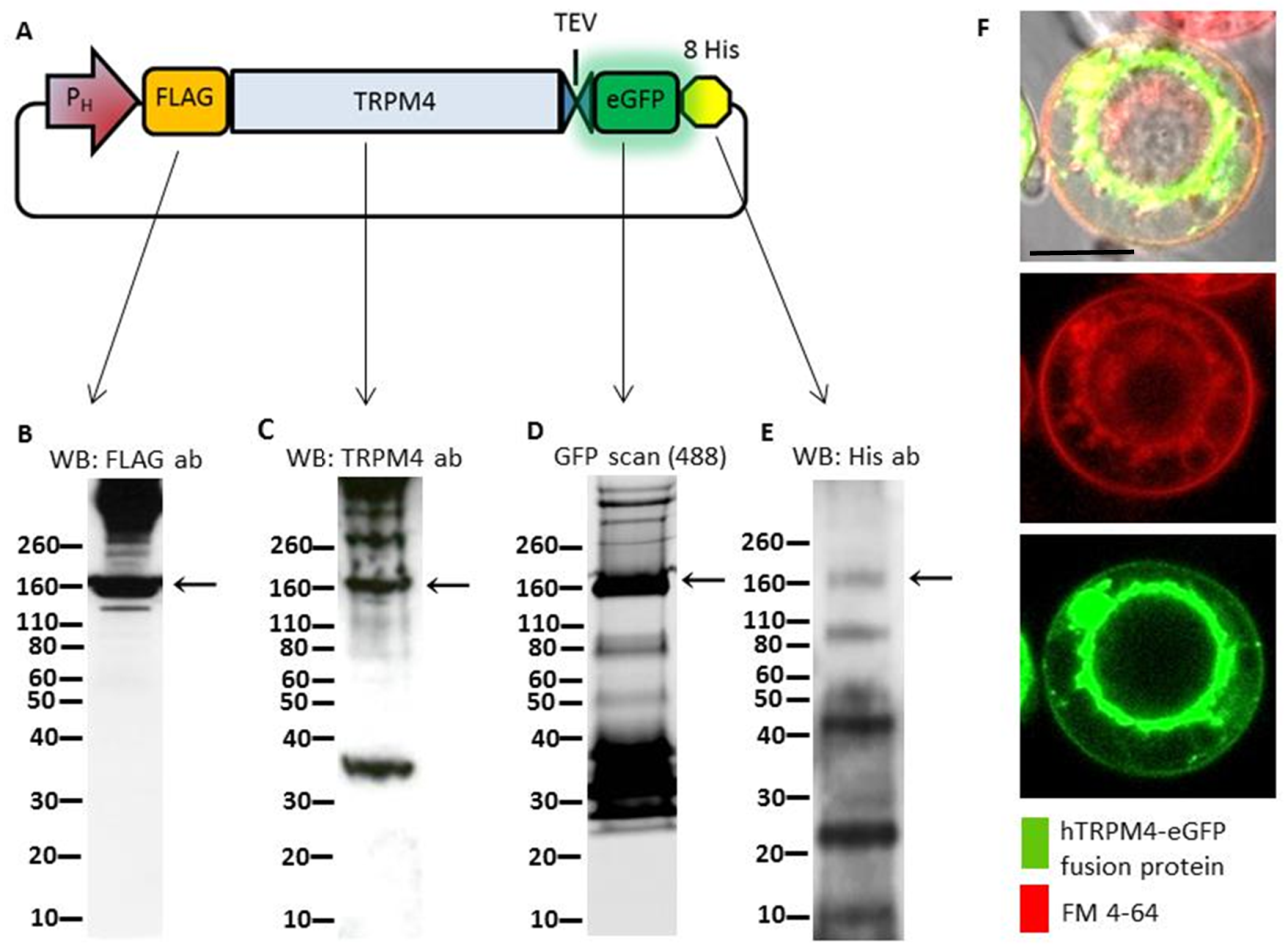


**Fig. S1.** Expression of FLAG-TRPM4-eGFP-8His fusion protein in Spodoptera frugiperda (Sf9) insect cells**.** (A) Diagram of FLAG-TRPM4-eGFP-8His pFastbac construct. (B) Lysates from TRPM4-eGFP fusion baculovirus-infected cells were fractionated by SDS-PAGE. Protein was immunoblotted and FLAG tag was detected. (C) Immunoblot with anti-TRPM4. (D) GFP scan of SDS-PAGE gel. (E) Immunoblot with anti-His. Arrows indicate the migration position (Mr 167kDa) of the full length TRPM4-eGFP fusion construct. (F) Confocal images of a baculovirus infected cell showing the distribution of hTRPM4-eGFP fusion protein (green channel, 488 laser line) and stained with FM4-64 membrane dye (red channel, 594 laser line). Top panel is an overlay of the green and red channels, the black scale bar represents 10µm. While the middle panel displays only the red channel and bottom panel the green channel.


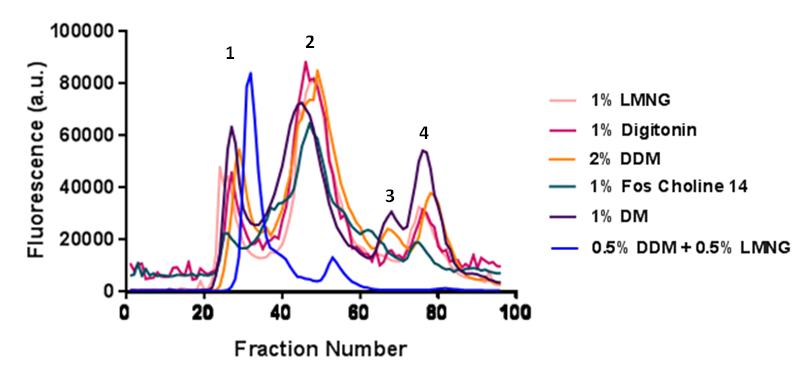


**Fig. S2.** **Overlaid FSEC profiles of the FLAG-hTRPM4-eGFP-8His fusion protein solubilised in different detergents.**  The FSEC elution profiles of FLAG-hTRPM4-eGFP-8His solublised in 1% LMNG, 1% digitonin, 2% DDM, 1% Fos-choline-14, 1% DM or 0.5% DDM + 0.5% LMNG are depicted in the line graph above, common peaks are labelled 1-4. Peak 1 is aggregated proten, Peak 2 is oligomeric protein, Peaks 3 and 4 contained truncated and/or degraded protein. The identity of the peaks was based on their elution retention time.


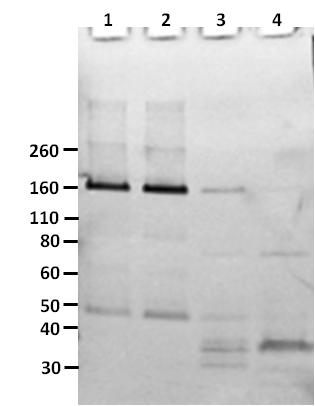


**Fig. S3. GFP scan of a SDS-PAGE gel with samples corresponding to peaks 1-4 from the FSEC profile of the FLAG-TRPM4-eGFP-8His fusion protein solubilised in 2% DDM.** All other detergents used for solubilisation of FLAG-TRPM4-eGFP-8His displayed similar bands for each peak. Lane 1 (peak 1 of figure 3.3) represents an aggregate peak as it elutes in the void volume, lane 2 (peak 2 of figure 3.3) represents an oligomeric protein peak, lane 3 (peak 3 of figure 3.3) represents a mixture of monomeric TRPM4, degraded protein and/or truncated proteins, lane 4 (peak 4 of figure 3.3) represents degraded protein and/or truncated protein/s.


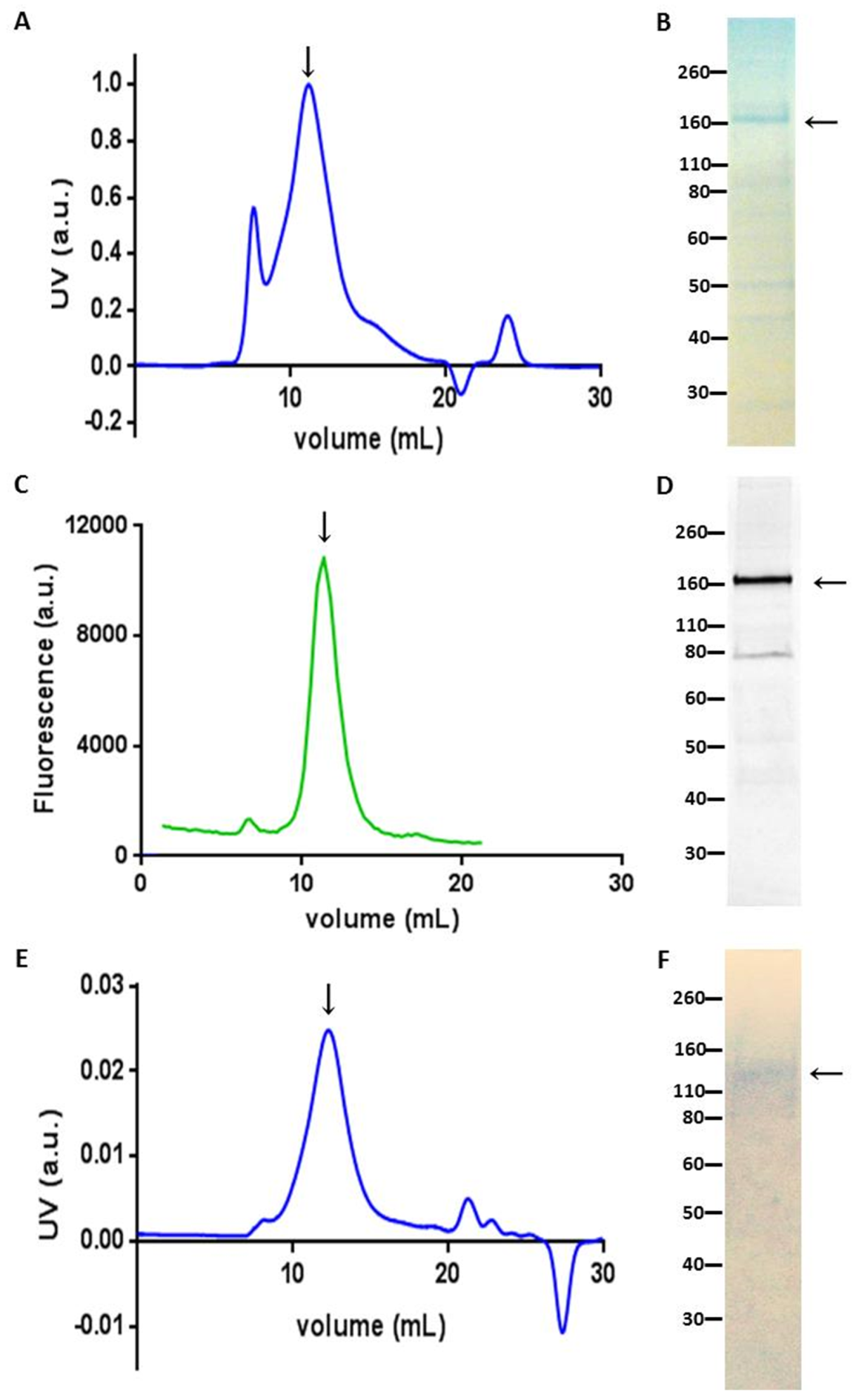


**Fig. S4. Purified FLAG-TRPM4-eGFP-8His fusion protein.** (A) UV chromatogram of the second round of size-exclusion chromatography purified hTRPM4-eGFP in DDM. (B) Coomassie-stained SDS-PAGE gel of the size exclusion chromatography purified hTRPM4-eGFP protein in DDM. (C) Fluorescence (488nm) chromatogram of the second round of size-exclusion chromatography purified hTRPM4-eGFP in DDM. (D) eGFP scan (473nm) of an SDS-PAGE gel with size exclusion chromatography purified hTRPM4-eGFP in DDM. (E) UV chromatogram of the second round of size-exclusion chromatography purified and TEV protease cleaved hTRPM4 in amphipol A8-35. (F) Coomassie-stained SDS-PAGE gel of the size exclusion chromatography purified cleaved hTRPM4 protein in amphipol A8-35.

**Fig. S5. Comparison of raw images from TEM of TRPM4 to class averages of cryo-EM and TEM images of TRPV1.** Images of TRPV1 in the left column are adapted from Liao and colleagues [^2^](#_ENREF_2)^,^[^3^](#_ENREF_3). Images in the right column contain raw TEM data of TRPM4 from Figure 11. (A) Left; 3-dimensional reconstruction of the top down view of TRPV1 from single particle cryo-EM data (Fig. 1F)[^2^](#_ENREF_2), right; TRPM4 TEM raw data displaying the fourfold symmetry. (B) Left; 2D class average of TRPV1 bottom view from cryo-EM data (Extended Fig 3C)[^3^](#_ENREF_3), right; TRPM4 TEM raw data. (C) Left; 2D class average of TRPV1 side view from cryo-EM data (Extended Fig 1C)[^3^](#_ENREF_3), right; TRPM4 TEM raw data. (D) Left; 2D class average of TRPV1 cryo-EM particles (Extended Fig 4E)[^2^](#_ENREF_2), right; TRPM4 TEM raw data image depicting the electron-lucent pore. (E) Left; 2D class average of TRPV1 cryo-EM particles (Extended Fig 1C)[^3^](#_ENREF_3), right; TRPM4 TEM raw data. (F) Left; 2D class average of TRPV1 cryo-EM particles (Extended Fig 1C)[^3^](#_ENREF_3), right; TRPM4 TEM raw data. (G) Left; 2D class average of TRPV1 cryo-EM particle images from the side (Extended Fig 1D)[^3^](#_ENREF_3), right; TRPM4 TEM raw data.


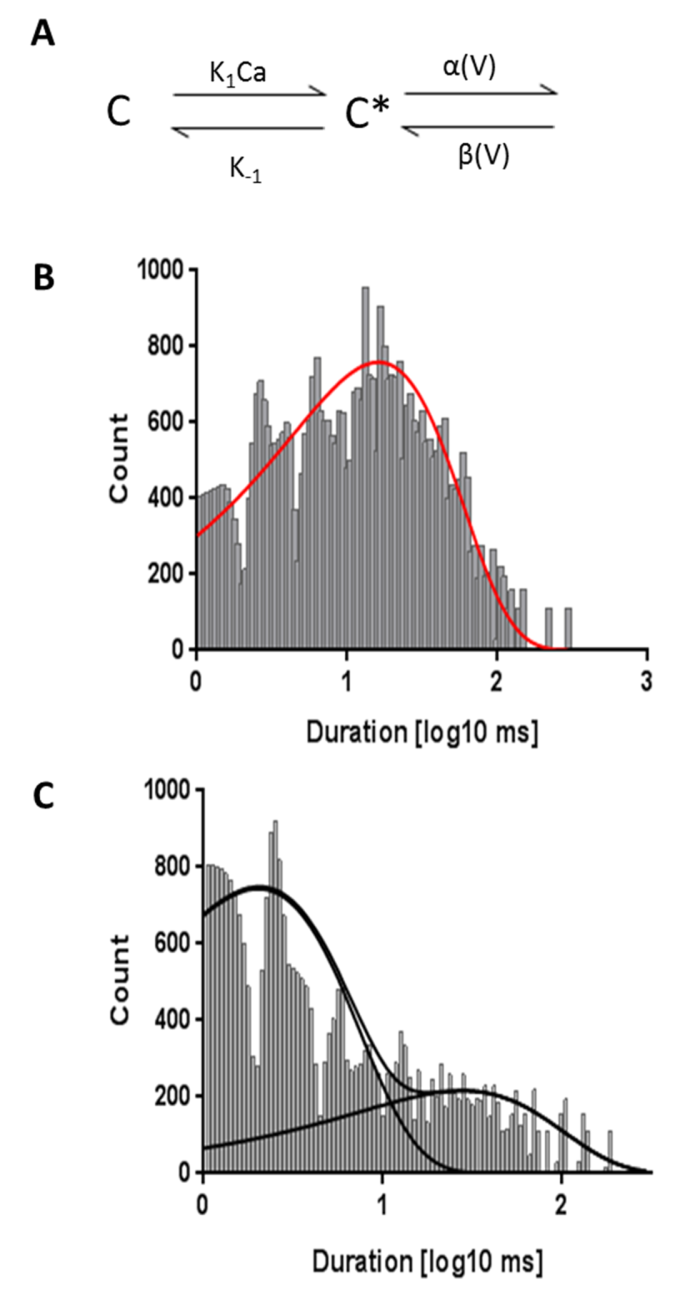


**Fig. S6.** Single channel modelling of the hTRPM4 fusion protein. (A) Minimal kinetic model to describe the Ca^2+^ and voltage-dependent gating of TRPM4 adapted from Nilius et al. [^4^](#_ENREF_4). Modelling the channel activity of hTRPM4 fusion protein using QuB software (University of Buffalo) indicated that the channel has (B) one open state and (C) two closed states.

**1 Digman, M. A., Caiolfa, V. R., Zamai, M. & Gratton, E. The Phasor Approach to Fluorescence Lifetime Imaging Analysis. *Biophysical Journal* 94, L14-L16, doi:**[**http://dx.doi.org/10.1529/biophysj.107.120154**](http://dx.doi.org/10.1529/biophysj.107.120154) **(2008).**

**2 Liao, M. F., Cao, E. H., Julius, D. & Cheng, Y. F. Structure of the TRPV1 ion channel determined by electron cryo-microscopy. *Nature* 504, 107-+, doi:10.1038/nature12822 (2013).**

**3 Cao, E., Liao, M., Cheng, Y. & Julius, D. TRPV1 structures in distinct conformations reveal activation mechanisms. *Nature* 504, 113-118, doi:10.1038/nature12823 (2013).**

**4 Nilius, B., Prenen, J., Janssens, A., Voets, T. & Droogmans, G. Decavanadate modulates gating of TRPM4 cation channels. *Journal of Physiology-London* 560, 753-765, doi:10.1113/jphysiol.2004.070839 (2004).**
